# Development of the Corticospinal and Callosal Tracts from Extremely Premature Birth up to 2 Years of Age

**DOI:** 10.1371/journal.pone.0125681

**Published:** 2015-05-08

**Authors:** Rodrigo M. Braga, Elise Roze, Gareth Ball, Nazakat Merchant, Nora Tusor, Tomoki Arichi, David Edwards, Daniel Rueckert, Serena J. Counsell

**Affiliations:** 1 The Computational, Cognitive and Clinical Neuroimaging Laboratory, Division of Brain Sciences, Hammersmith Hospital Campus, Imperial College London, London, United Kingdom; 2 Center for Brain Science, Harvard University, Cambridge, Massachusetts, United States of America; 3 Athinoula A. Martinos Center for Biomedical Imaging, Department of Radiology, Massachusetts General Hospital & Harvard Medical School, Charlestown, Massachusetts, United States of America; 4 Wilhelmina Children’s Hospital, University Medical Center Utrecht, University of Utrecht, the Netherlands; 5 Centre for the Developing Brain, Division of Imaging Sciences and Biomedical Engineering, King's College London, London, SE1 7EH, United Kingdom; 6 Department of Computing, Imperial College London, South Kensington Campus, London, United Kingdom; Duke-NUS Graduate Medical School, SINGAPORE

## Abstract

White matter tracts mature asymmetrically during development, and this development can be studied using diffusion magnetic resonance imaging. The aims of this study were i. to generate dynamic population-averaged white matter registration templates covering in detail the period from 25 weeks gestational age to term, and extending to 2 years of age based on DTI and fractional anisotropy, ii. to produce tract-specific probability maps of the corticospinal tracts, forceps major and forceps minor using probabilistic tractography, and iii. to assess the development of these tracts throughout this critical period of neurodevelopment. We found evidence for asymmetric development across the fiber bundles studied, with the corticospinal tracts showing earlier maturation (as measured by fractional anisotropy) but slower volumetric growth compared to the callosal fibers. We also found evidence for an anterior to posterior gradient in white matter microstructure development (as measured by mean diffusivity) in the callosal fibers, with the posterior forceps major developing at a faster rate than the anterior forceps minor in this age range. Finally, we report a protocol for delineating callosal and corticospinal fibers in extremely premature cohorts, and make available population-averaged registration templates and a probabilistic tract atlas which we hope will be useful for future neonatal and infant white-matter imaging studies.

## Introduction

Premature birth is increasingly prevalent and survival rates have been steadily improving [1,2,3,4]. However, infants born preterm remain at high risk of developing neurological impairments in later life [5,6]. Diffusion tensor imaging (DTI) [7] is a magnetic resonance imaging (MRI) tool that provides objective metrics such as fractional anisotropy (FA) and mean diffusivity (MD) which reflect underlying tissue structure and allow brain development to be assessed non-invasively [8,9,10]. As white matter matures, the axon fibers become increasingly cohesive, water content decreases and myelination commences [11]. These cytoarchitectural changes constrain the free diffusion of water along directions perpendicular to the axon fibers, which increases FA and decreases MD [12].

White matter undergoes dramatic changes during early life. Before term, rapid and linear increases in FA can be observed in white matter [10,13,14,15,16]. These sharp changes continue beyond term-equivalent age (TEA), such that by 1 year of age term-born infants have close to full white matter maturation [9]. Beyond 1 year of age, the rate of change of anisotropy plateaus, and more gradual changes in FA can be observed [17,18] which can continue well into adolescence [16].

Premature infants typically display delayed white matter maturation when compared to term-born controls [8,9,19,20,21,22,23]. Reduced white matter anisotropy in infancy has been shown to predict neurological outcome in later life [24,25,26]. Reduced tract volume of and FA values in callossal fibres at term-equivalent age (TEA) is associated with cognitive impairment at 2 years of age [24]. Both the genu and splenium of the corpus callosum show impaired development following preterm birth [24], with some evidence that posterior callosal fibers are more affected than anterior ones [21,27,28]. Similarly, abnormalities in anisotropy within the internal capsules and corticospinal tracts are indicative of motor impairments in later life [25,29]. These findings could be important in informing future diagnostic and preventative clinical strategies. Studying white matter development at a finer temporal resolution may help explain why certain tracts are compromised while others are spared.

The aims of this study were i. to generate dynamic population-averaged white matter registration templates for preterm infants covering in detail the period from 25 weeks gestational age (GA) to term, and extending to 2 years of age based on DTI and fractional anisotropy, ii. to produce tract-specific probability maps of the corticospinal tracts, forceps major and forceps minor using probabilistic tractography, and iii. to assess the development of these tracts throughout this critical postnatal period of neurodevelopment.

## Materials and Methods

Ethical permission for MR imaging was granted by the Hammersmith Hospital Research Ethics Committee. Written parental consent was obtained prior to imaging for each subject.

### Subjects

The study group (Table 1) included 99 infants (61 female) who were born at median 27.7 (24.9–34.3) weeks GA with median birth weight 1.07 (0.670–2.05) kg. Fourteen infants were scanned twice and 2 were scanned 3 times.

**Table 1:** Age group demographics.

Age Group	n	Sex (females)	GA (wks)	PMA at scan (wks)	Weight at scan (kg)	HC at scan (cm)
25–28 wks PMA	9	6	26 (24.9–26.6)	27.7 (25.3–28.6)	0.78 (0.58–0.84)	23.5 (21.5–24)
29–30 wks PMA	15	9	26.7 (24.6–29.7)	29.4 (29–30.9)	0.98 (0.8–1.3)	24.6 (23.5–27.5)
31–32 wks PMA	15	11	28.4 (26.1–31.9)	32 (31.1–32.7)	1.24 (1.0–1.8)	27.3 (26–30)
33–35 wks PMA	15	6	30.4 (26–31.4)	33.4 (33–35.3)	1.72 (1.2–2.6)	30.1 (28.2–32.4)
TEA	15	10	28.7 (24.6–31.9)	40.6 (39.3–42.1)	2.98 (1.8–4.4)	35 (32–37.5)
1 year	15	11	27.3 (25.7–32.7)	96 (92–104)	9.52 (7.6–10.2)	46.3 (43.2–48.3)
2 years	15	8	27.3 (24.7–34.3)	151 (144–174)	11.06 (10.2–17.4)	48.5 (45.7–53.3)

Values are median (range). TEA: term-equivalent age; GA: gestational age; wks: weeks; PMA: post-menstrual age; HC: head circumference.

Images were selected from a database of 435 images obtained between September 2006 and June 2010, which were acquired as part of an on-going study investigating preterm brain development. Inclusion criteria for this study were i. preterm birth < 37 weeks GA ii. no evidence of focal abnormality on conventional MRI, iii. DTI data free from artefact due to patient motion, and iv. developmental quotient scale ≥85 obtained using either Griffiths scales [30] or Bayleys III assessment [31] at a corrected age > 1 year.

Subjects were divided into 7 age groups based on their post-menstrual age (PMA) at scan; 25–28 weeks PMA, 29–30 weeks PMA, 31–32 weeks PMA, 33–35 weeks PMA, term equivalent age (39–42 weeks PMA), 1 year corrected age (11–14 months) and 2 years corrected age (22–29.5 months). The first 15 infants (alphabetically) to be imaged in each age group who fulfilled the inclusion criteria were selected for inclusion in this study. Despite a large dataset of preterm brain images we did not acquire 15 datasets that were free of motion, focal lesions and DQ scores ≥ 85 for the age range 25–28 weeks GA. This group contained 9 subjects.

### Magnetic resonance imaging

MRI was performed on a Philips 3-Tesla system (Philips Medical Systems, Netherlands) using an 8-channel phased array head coil. The 3D-MPRAGE and high-resolution T2-weighted fast spin echo images were obtained before diffusion tensor imaging. Single-shot EPI DTI was acquired in the transverse plane in 32 non-collinear directions using the following parameters: repetition time (TR): 8000 ms; echo time (TE): 49 ms; slice thickness: 2 mm; field of view: 224 mm; matrix: 128 × 128 (voxel size: 1.75 × 1.75 × 2 mm^3^); *b* value: 750 s/mm^2^. Data were acquired with a SENSE factor of 2.

All examinations were supervised by a pediatrician experienced in MRI procedures. Infants were sedated with oral chloral hydrate (25–50 mg/kg) prior to scanning and pulse oximetry, temperature and electrocardiography data were monitored throughout. Ear protection was used for each infant, comprising earplugs moulded from a silicone-based putty (President Putty, Coltene Whaledent, Mahwah, NJ) placed in the external ear and neonatal earmuffs (MiniMuffs, Natus Medical Inc, San Carlos, CA).

DTI analysis was performed using FMRIB's Diffusion Toolbox (FDT v2.0) as implemented in FMRIB's Software Library (FSL v4.1; www.fmrib.ox.ac.uk/fsl) [32]. Each infant's diffusion weighted images were registered to their non-diffusion weighted (*b*0) image and corrected for differences in spatial distortion due to eddy currents. Non-brain tissue was removed using the brain extraction tool (BET)[33]. Diffusion tensors were calculated voxel wise, using a simple least squares fit of the tensor model to the diffusion data. From this, the tensor eigenvalues and FA maps were calculated.

### Tractography protocols

Tractography was carried out using FMRIB's Diffusion Toolbox (FDT)[34]. Fibre tracking protocols were adapted from the approach described by Wakana et al [35] using the *b*0 image, FA map and color-coded FA map to locate the relevant anatomy. One postgraduate student delineated the seed and waypoint masks manually in all infants and the intraobserver variability was assessed on a pilot dataset of 5 subjects. Seed and waypoint mask sizes for each tract and age group (Table 2) were determined by first delineating optimal masks in 5 subjects per age group, based on their anatomy. The modal mask size was then chosen for tractography in the whole age group. Connectivity distributions were generated from every voxel in the seed mask, and only those paths that went through the waypoint masks were retained. The distributions were then normalized by dividing by the number of samples going from the seed mask through the waypoints, controlling for differences in mask size used [36]. The resulting connectivity distributions were thresholded at the 1% probability level. For the forceps major and forceps minor, fiber tracking was conducted twice for each subject, first from left to right and then from right to left. This is because the probability distribution are biased towards streamlines that cross the waypoint mask. Distal streamlines (i.e. those heading in the opposite direction to the waypoint mask) are not adequately represented. The two streamline distributions were normalised individually then added together and averaged.

**Table 2:** Mask sizes (in number of voxels) used for tractography of corticospinal tracts (CST), forceps minor (FCmin) and major (FCmaj) in each age group.

Age Group		CST		FCmin	FCmaj
	CP	PLIC	CS		
25–28 wks PMA	12	14	n/a	20	40
29–30 wks PMA	12	15	n/a	28	54
31–32 wks PMA	15	17	n/a	32	70
33–35 wks PMA	15	17	n/a	36	70
TEA	17	19	n/a	72	108
1 year	21	21	n/a	84	165
2 years	21	21	n/a	84	165

Mask sizes were determined by delineating anatomically optimal masks in 5 subjects from each age group, then using the modal mask size for the entire group. The central sulcus mask (CS) did not have a set number of voxels per age group, because both pre- and post-central gyri were delineated in each subject using as many voxels as necessary. wks GA: weeks gestational age; CP: cerebral peduncle; PLIC; posterior limb of the internal capsule.

### Corticospinal tracts

**Fig 1:**
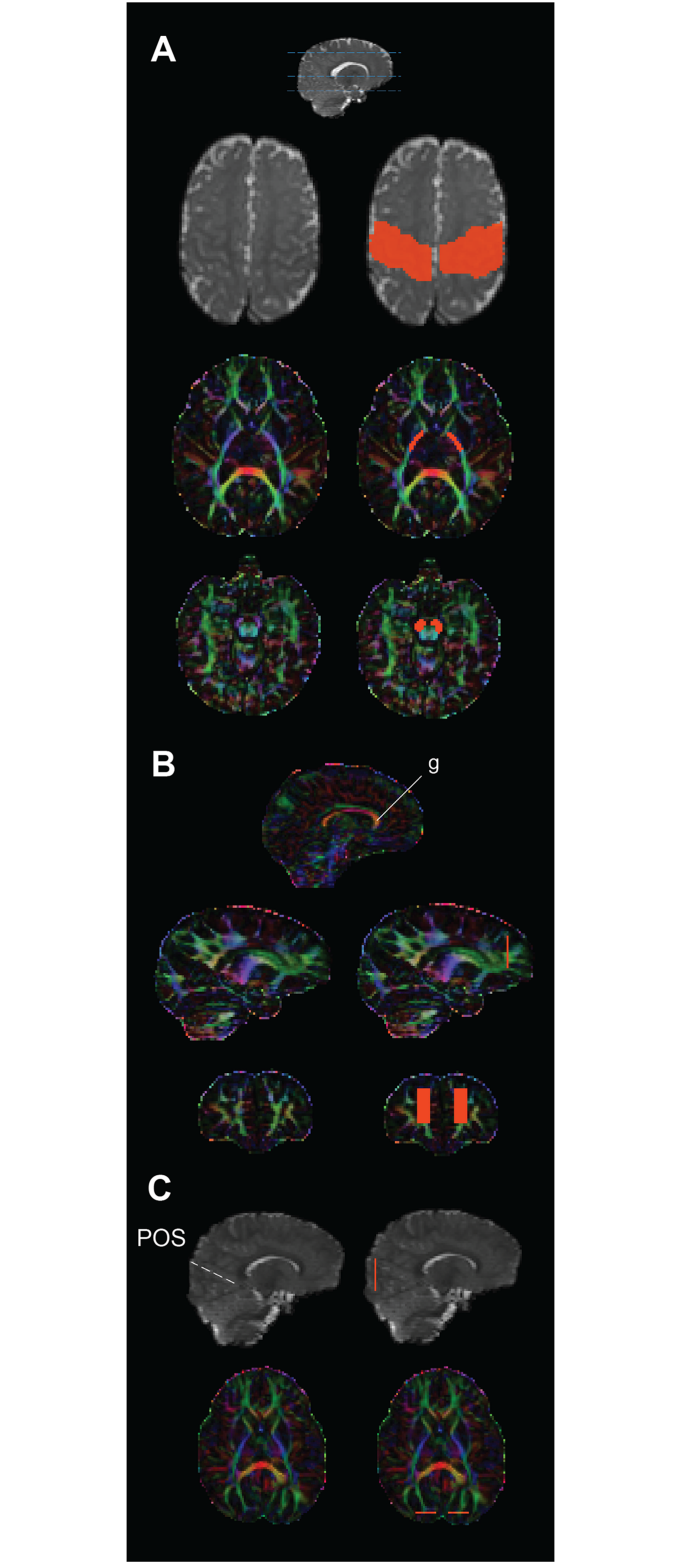
Anatomical regions of interest (in orange, right column in each) used to guide tractography of the a) corticospinal tracts, b) forceps major, and c) forceps minor in one infant overlayed on their B0 image (grey) and v1 colour map.

We focused specifically on cerebral portions of the CSTs, beginning at the midbrain and extending to the gyri surrounding the central sulcus of each hemisphere. A seed mask was drawn around the cerebral peduncle (see Table 2 for mask sizes in each age group) on the color-coded FA map at the level of the decussation of the superior cerebellar peduncles (Fig 1). Two waypoint masks were added to ensure the tractography adhered to fibers of the CSTs. The first was placed around the posterior limb of the internal capsule (PLIC), in the most inferior slice where both the anterior and posterior limbs could be clearly seen in the FA map and color coded FA map. A second waypoint was drawn using the *b*0 image to include the pre-central and post-central gyri on the axial slice that was mid-way between the top of the lateral ventricles and the superior cortical surface. Even in the youngest age group the precentral and postcentral gyri can be identified surrounding the central sulcus on a structural image. By using the central sulcus as a reference, this protocol could therefore be used for all age groups despite differences in cortical folding.

### Forceps Minor (FCmin)

A coronal slice was selected halfway between the most anterior aspect of the corpus callosum and the anterior tip of the frontal lobe. Although Wakana and colleagues [35] delineated the entire cortex at this slice as a region of interest (ROI) in the adult brain, this same protocol failed to produce reliable tracts in the neonatal brain. Therefore, smaller ROIs were delineated using the V1 colour map to include the frontal white matter that can be clearly seen on coronal slices (Fig 1). Rectangular ROIs were placed starting at the inferior boundary of the genu and extending to include the superior white matter visible at that slice, while excluding peripheral cortical voxels.

### Forceps Major (FCmaj)

As described by Wakana *et al*. [35] two ROIs were positioned in the most posterior slice at which the parieto-occipital sulcus could be seen, however smaller rectangular ROIs were again used that extended only into the occipital lobe but excluded lateral and ventral cortical voxels (Fig 1).

### Exclusion masks

Tractography of the forceps sometimes resulted in fibers that crossed the midline at nonsense regions (i.e. where there is no anatomical connection between the hemispheres), particularly in the most immature infants where the midline separation distance approached the voxel resolution. In order to prevent midline crossings, a mask through the midline was used as an exclusion mask. In 2 subjects, tracking of the FCmin resulted in projections to posterior regions via the fronto-occipital fasciculus. Although these are not nonsense projections these fibers do not form part of the FCmin and their inclusion was avoided by using a full coronal slice halfway down the anterior-posterior axis as a second exclusion mask.

### Tract indices

Thresholded tracts were binarised and tract-wise volume, mean FA and MD values were determined by taking the average for all voxels included in each binarised tract.

### Statistical analysis

We explored whether FA, MD and volume displayed different rates of change across each tract in the neonatal cohort (subjects with corrected age at scan < 50 wks GA). FA, MD and volume were entered as the dependent variable in a linear regression, with corrected age at scan (CAS; as covariate), tract type (as fixed factor) and the interaction of CAS and tract type as the independent variables. Analysis of covariance (ANCOVA) was then performed for FA, MD and volume for all tracts, and pairwise parameter contrasts explored tract-wise differences in the rate of change of each dependent variable.

### Atlas generation

**Fig 2:**
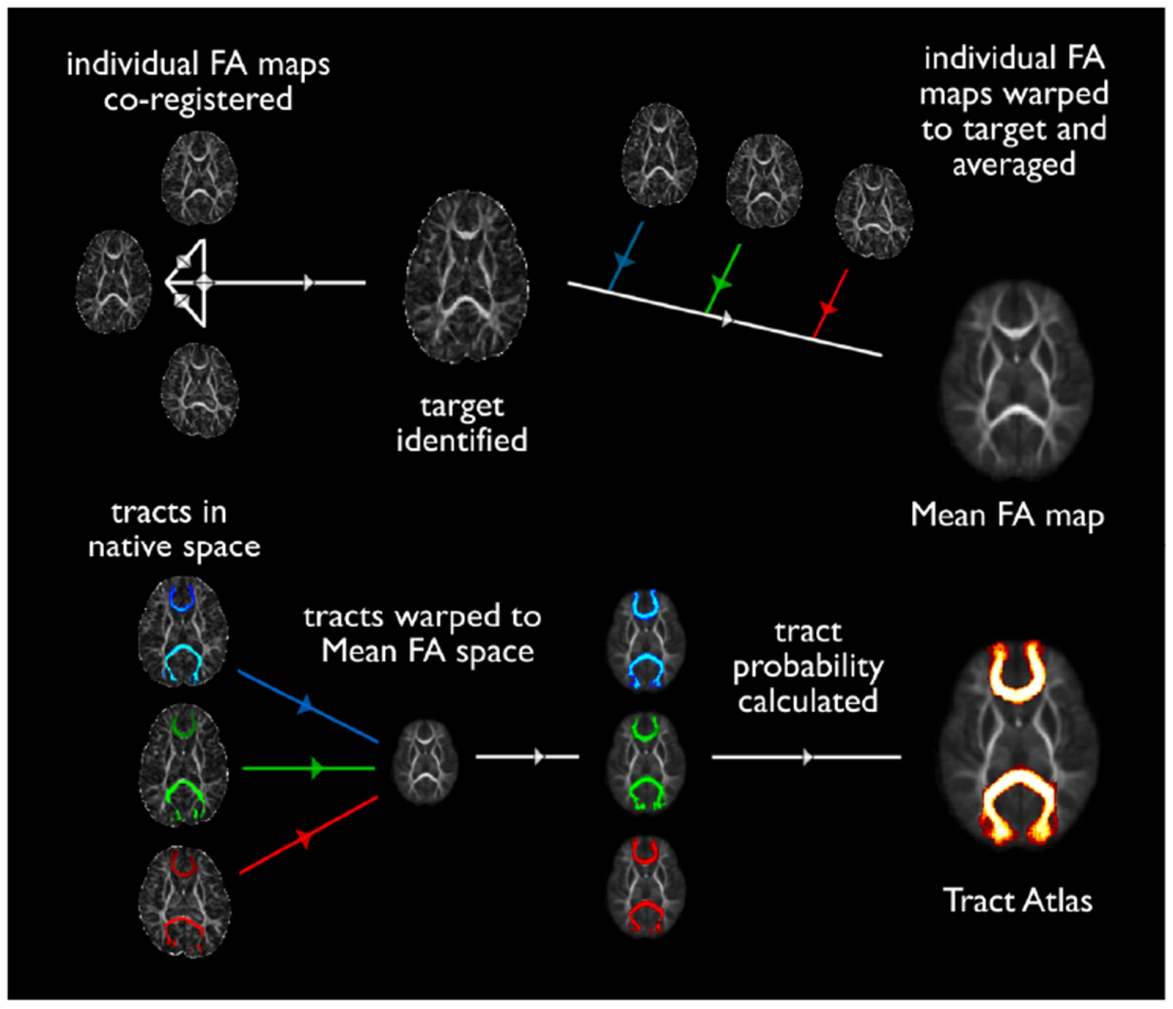
Schematic of registration steps taken to produce probabilistic FA maps (a) and tract atlases (b) in each age group. The target image was identified as that which required the least transformation to register with all others in its age group. Once the individual FA images in each age group were co-registered, FA values at each voxel were averaged together to produce a population-mean FA map for each time point. Tractography distributions from each subject were binarised and coregistered using the same warp matrix from the mean FA atlas. The number of tractography distributions passing through each voxel (i.e. the overlap across subjects) was then computed to represent the degree of conservation each tract across the population (Tract Atlas).

FA and tract distribution maps from each subject were coregistered to generate seven age-specific population-averaged atlases. Image registration was performed using tract-based spatial statistics (TBSS) as described in Smith *et al*. [37] and optimised for neonates in Ball *et al*. [23]. Briefly, within an age group each subject’s FA map was warped to all other subjects’ FA maps. Through this the ‘most typical’ subject was identified (i.e. the subject whose FA map required the least warping to be aligned with all others). This subject became the target image for that age group, and all FA maps were registered to this target. The mean FA atlas was then generated by taking the mean FA value at each voxel. Binarised tractography distributions from each subject were registered to the mean FA atlas using the same warp matrix and the overlap probability was used to create the population-averaged tract distribution atlas (see Fig 2).

## Results

### Neurodevelopmental assessments

The median DQ of the infants was 98 (87–116) for those infants who were assessed using the Griffiths Developmental Scales and 95 (89–124) for those infants assessed using the Bayleys III. The median age at assessment was 25 (18–31) months for the Griffiths and 25.5 (15.5–27) months for the Bayleys III.

### Reproducibility

The tractography protocol was performed twice on 5 infants at term-equivalent age and the coefficient of variance was found to be 0.4% for tract mean FA and 1.5% for volume measurements.

### Mean FA and tract probability atlases

**Fig 3:**
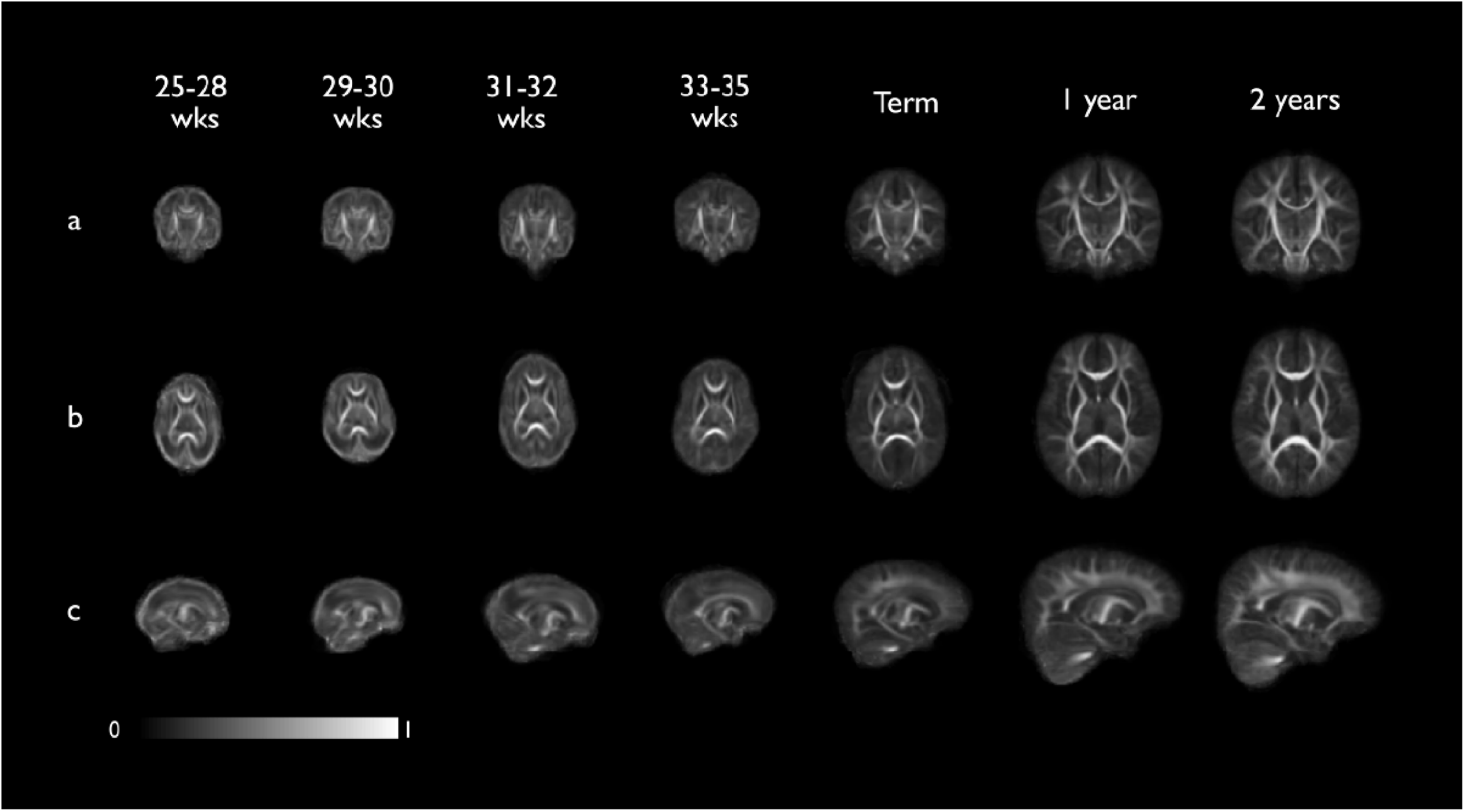
Coronal (a), axial (b) and sagittal (c) views of the seven population-averaged fractional anisotropy (FA) atlas at the 7 developmental time points. Intensity bar indicates FA value. These registration templates are freely available at www.brain-development.org.

**Fig 4:**
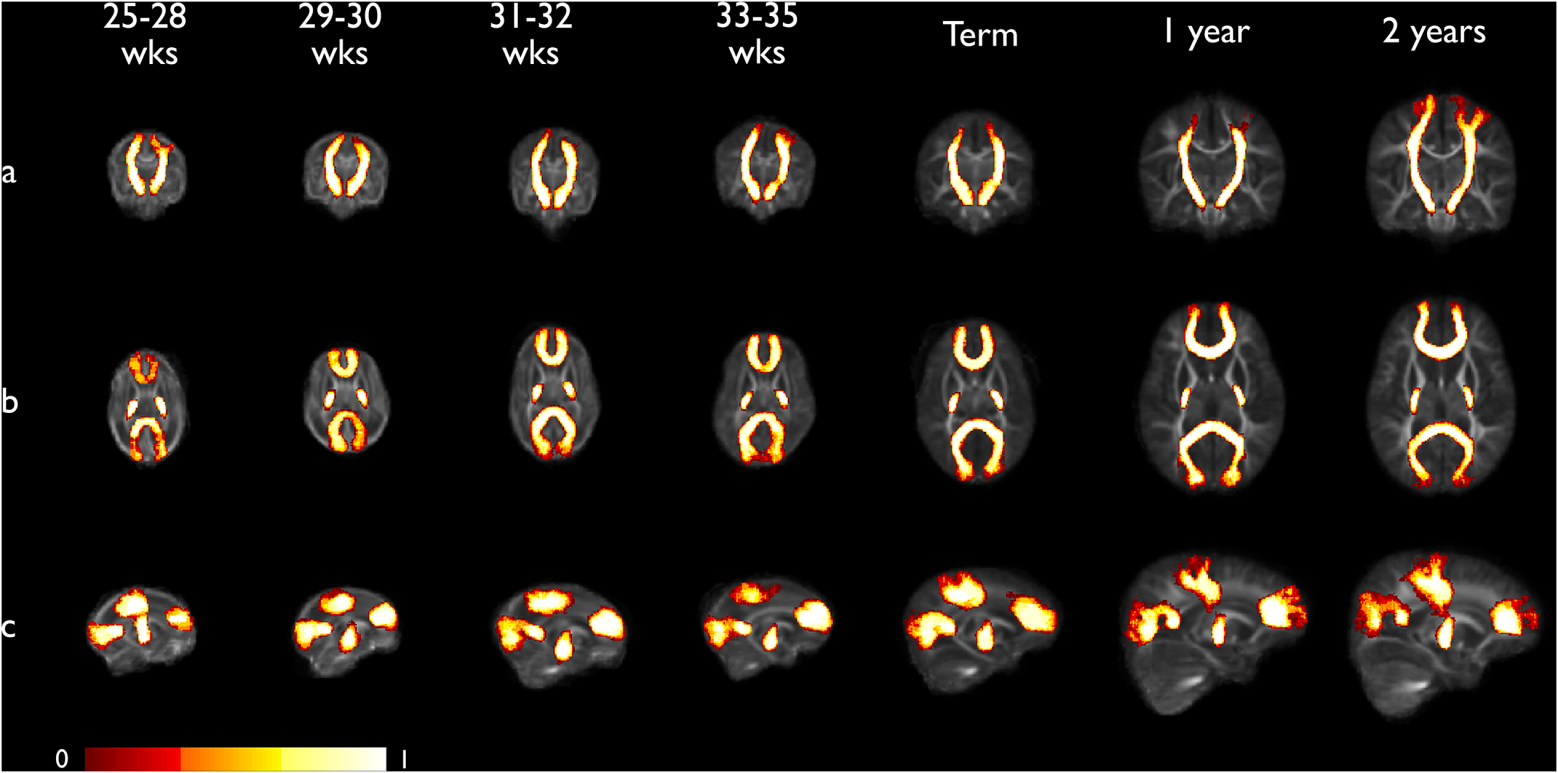
Coronal (a), axial (b) and sagittal (c) views of population-averaged tract probability maps for each developmental time point overlaid on mean FA atlas. Corticospinal tracts (CST), forceps minor and major are shown, thresholded at the 0.25 probability level. The intensity of each voxel represents the proportion of tracts that pass through that voxel across the whole group. This ranges from 0 probability (tract present at this voxel in none of the subjects) to 1 (tract present at this voxel in all subjects). The maximum spatial variation across the group is represented, however information is retained on the distributions that are most conserved across the population.

Figs 3 and 4 show the mean FA and tract probability atlases for each developmental time point.

### Diffusion indices

**Fig 5:**
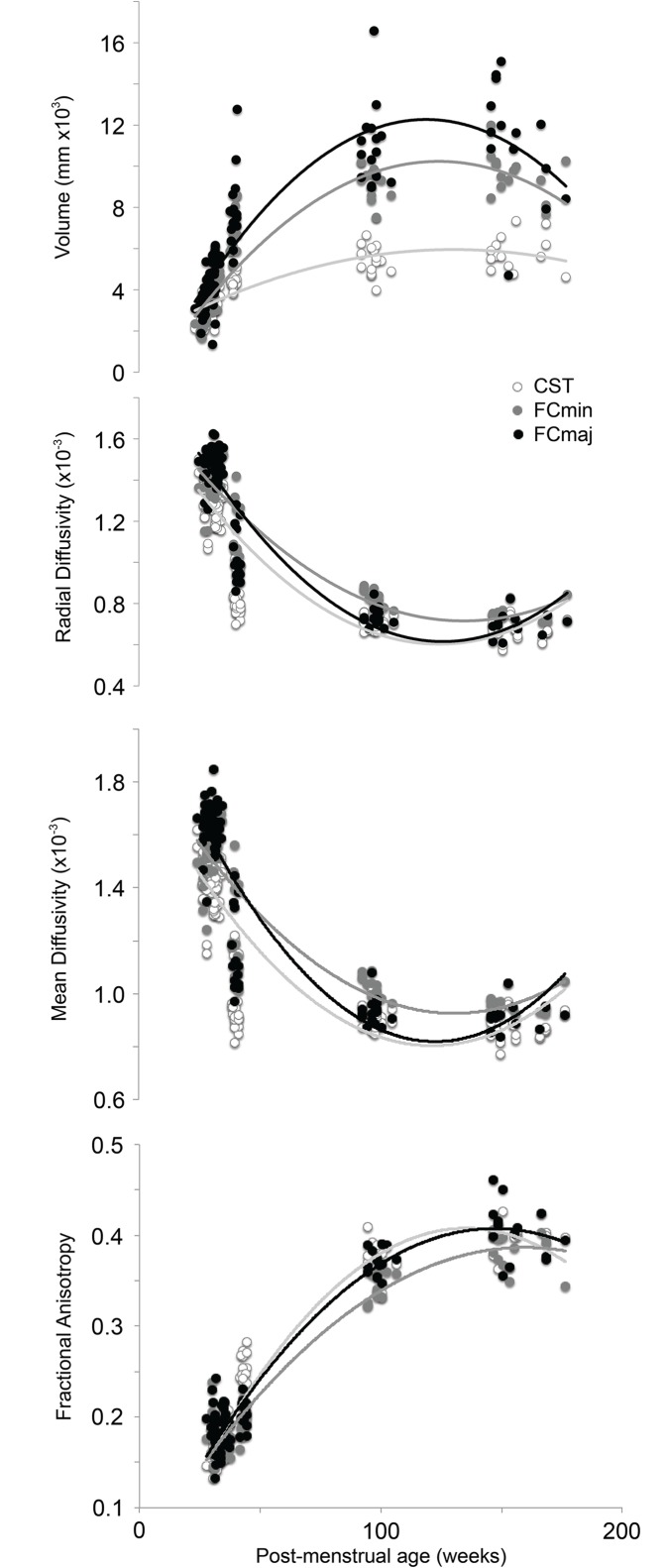
Group-wise developmental changes in fractional anisotropy (FA), axial (L1) and mean diffusivity (MD) and the length of the two remaining eigenvectors (L2 and L3) taking place between viable birth (25 weeks gestational age; wks GA) to 2 years corrected age (CA). CST: corticospinal tracts; FCMIN: forceps minor; FCMAJ: forceps major. X-axis is non-linear and chosen to display the variation across the atlas timepoints.

Fig 5 shows developmental changes in volume, FA, and mean and radial diffusivity on a tract-by-tract basis. Each variable displayed a non-linear relationship with age at scan, with the rate of change decreasing rapidly as age at scan increased. Quadratic trend lines were a better fit to the data than linear trends (Table 3). Volume and FA increased, while radial and mean diffusivity decreased, with increasing age at scan. The changes observed could generally be described in two stages; 1) rapid changes from birth until 1 year of age (range; FA: 0.13–0.41, MD: 0.0018–0.00081, RD: 0.0016–0.00066, volume: 1,400–17,000mm^3^), and 2) a levelling off from 1 to 2 years of age. Between 1 and 2 years of age, changes in FA and MD were markedly lower (range; FA: 0.32–0.45, MD: 0.0011–0.00078, RD: 0.00088–0.00058, volume: 4,000–17,000mm^3^).

**Table 3:** R squared values for quadratic and linear lines of best fit for the whole study population (all ages).

	Volume		RD		MD		FA	
	linear	quadratic	linear	quadratic	linear	quadratic	linear	quadratic
CST	0.606	0.696	0.627	0.761	0.564	0.701	0.842	0.945
FCmin	0.712	0.863	0.731	0.824	0.665	0.762	0.906	0.943
FCmaj	0.594	0.763	0.712	0.853	0.656	0.803	0.875	0.953

Quadratic values consistently provided a better fit to the data, suggesting that developmental changes were not linear in infancy.

**Fig 6:**
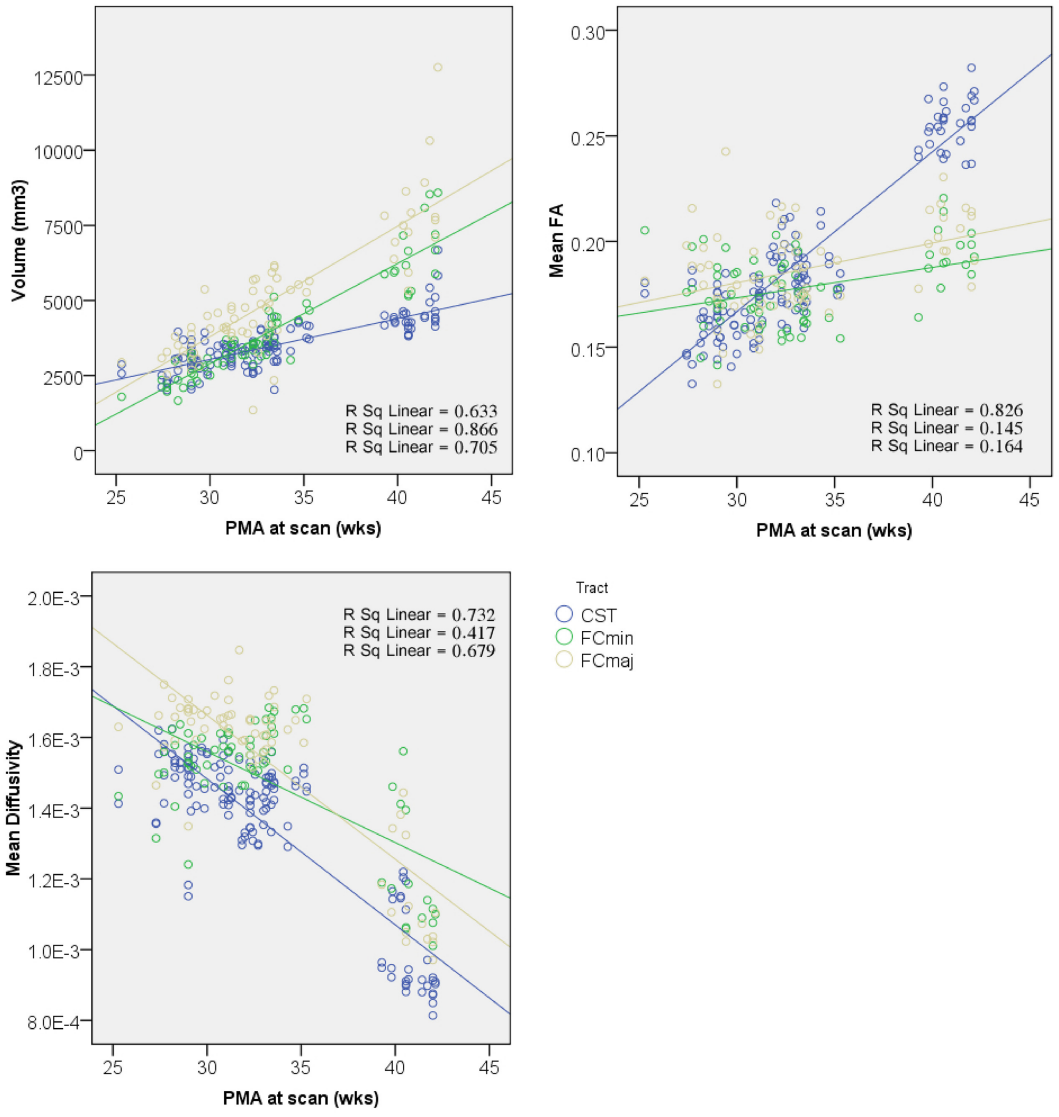
Differences in rate of white matter maturation during preterm period shown in corticospinal (CST, blue) and forceps minor (FCmin, green) and forceps major (FCmaj, yellow). CSTs displayed faster rate of change of FA and MD, possibly due to an earlier onset of myelination [39], whereas callosal fibers exhibited faster volumetric growth. PMA; post-menstrual age, FA; fractional anisotropy, MD; mean diffusivity.

Fig 6 focuses on the preterm period, and shows differences across tracts in the rate of change of FA, MD and volume over time. These changes could be approximated linearly and were therefore investigated further using linear regression.

### Statistical analysis

Across all tracts, the ANCOVA revealed a main effect of both CAS and tract type on FA (CAS: F_1,268_ = 359.0, tract type: F_3,268_ = 48.8, all p < 0.001), MD (CAS: F_1,268_ = 488.6, p < 0.001, tract type: F_3,268_ = 4.5, p < 0.005) and volume (CAS: F_1,268_ = 666.0, tract type: F_3,268_ = 30.5, all p < 0.001) indicating that overall each variable changed significantly with increasing age and at different rates within each tract. Additionally, a significant interaction was observed between CAS and tract type for FA (F_3,268_ = 54.3, p < 0.001), MD (F_3,268_ = 5.4, p < 0.002) and volume (F_3,268_ = 42.8, p < 0.001) suggesting that overall there were different rates of change in FA, MD and volume within different tract types. The F statistic for the linear model used was F_7,268_ = 78.8 (p < 0.001, adjusted R^2^ = 0.665) for FA, F_7,268_ = 88.0 (p < 0.001, adjusted R^2^ = 0.689) for MD, and F_7,268_ = 142.3 (adjusted R^2^ = 0.782) for volume.

Given that overall there was a significant interaction, we assessed whether the interaction was present in all the tracts by contrasting parameter estimates between pairs of tracts. Both the left CST and FCmaj were independently used as references in the contrasts. For FA, significant differences were observed between the FCmin and FCmaj vs. left CST (p < 0.001), but not between right vs. left CST (p = 0.554). Similarly, significant differences in the rate of change of FA were observed between the left and right CST vs. FCmaj (p < 0.001) but not between the FCmin vs. FCmaj (p = 0.846). For MD, significant differences were observed between the FCmin vs. left CST (p < 0.05), but not between right CST or FCmaj vs. left CST (both p > 0.1). When compared to the FCmaj, significant differences in rate of change of FA were observed between the FCmin vs. FCmaj (p < 0.002) but not between the left and right CST vs. FCmin (both p > 0.1). Finally, for volume, significant differences were observed between the FCmin and FCmaj vs. left CST (p < 0.001), but not between right vs. left CST (p = 0.806). Similarly, significant differences in rate of change of FA were observed between the left and right CST vs. FCmaj (p < 0.001) but not between the FCmin vs. FCmaj (p = 0.788).

## Discussion

In this study, we describe a reproducible tractography protocol to assess brain development in 3 major tracts; the corticospinal tract (left and right), forceps major and forceps minor at a high temporal resolution on a tract-by-tract, population-averaged basis. The present study used a tractographic approach which allows the quantitation of average diffusion values for each tract as a whole. Our reported diffusion values therefore represent changes in both deep central and peripheral white matter voxels. These changes can roughly be divided into two stages; 1) a rapid increase in anisotropy and volume between birth and term-equivalent age, and 2) a gradual levelling off between 1 to 2 years of age.

The whole-tract approach is particularly useful for comparing development across different tracts. The reported interactions between tract type and CAS in the ANCOVAs indicated that white matter volume and diffusion metrics changed asymmetrically across the tracts studied. When this interaction was explored further in the preterm period, the left and right CSTs were found to be showing similar rates of change in volume, MD and FA, whereas the callosal fibers showed a faster rate of volumetric growth and a slower rate of change in FA than the CSTs (Fig 6). This indicates that the left and right CSTs grow and mature at similar rates. In addition, the CSTs mature faster, but also grow less rapidly than the callosal fibers. The anterior and posterior callosal fibers showed similar rates of change in FA and volume. When change in MD was assessed, the FCmaj and left and right CSTsall showed similar rates of decrease, whereas the FCmin displayed a slower rate of decrease in MD. This might be taken as evidence to support a posterior to anterior gradient of development [38], where the FCmaj develop earlier than the more anterior FCmin.

In the preterm period, the rate of change of FA in the CSTs was markedly faster than the forceps. This may be because the CSTs undergo the first stages of myelination at around 34 weeks GA, whereas the callosal fibers do not myelinate until late in the third trimester, perhaps as late as 46 weeks PMA [39]. Although myelination is an important modulator of anisotropy in the brain, myelin itself is not necessary for anisotropy. Other factors, such as the cohesiveness and compactness of axonal membranes [12] and the level of water content of the brain [40] contribute to anisotropy and also exhibit changes during development. DTI studies over different gestational ages have shown decreases in MD values with increasing maturity [41,42, 43]. Studies in older children have shown that MD continue to decline throughout the first decade of post-natal life [44], and into young adulthood [45]. The brain exhibits a 14–18% reduction in water content from birth to adulthood [46] and increased binding of water to macromolecules such as myelin, leads to a decreased extracellular space which reduces separation of structures such as cell membranes, and restricts water molecular motion [12,41,47]. These maturational factors contribute to global trends in FA and MD, but not tract-specific developmental asymmetries. The changes we observe therefore more likely reflect tract-specific differences in local axonal and myelin maturity [12].

In this study, we included only apparently healthy but extremely preterm infants who had brain MR images without any focal changes of known pathologic importance. We also included only infants whose outcome was considered normal in terms of their developmental quotient between 1 and 2 years. Therefore, we consider these infants optimal within the limits of our ability to assess brain development near the age of viability. We accept the fact that the concept of normality in this extremely preterm population is relative and that they may have some neurodevelopmental problems that become apparent only at an older age. Within these caveats, our DTI data may be representative of normal findings. Complimentary research using fetal cohorts may shed further light on developmental differences between preterm and term-born infants. Knowledge of the changes in diffusion metrics in major white matter tracts during development in such preterm infants compliments qualitative imaging evaluation and is essential to the assessment of MR images in this group of infants. Future studies should replicate these findings in different centres and scanners to determine to what extent the population-averaged diffusion metrics described here can be generalized.

We generated population-averaged tractography atlases in normally developing preterm children including seven mean FA maps to be used as registration templates for future studies looking at white matter development, and probabilistic maps of the three major white matter tracts studied. Other studies have generated comprehensive tractography atlases in preterm neonates at close to term equivalent age [48], term infants and children [49,50] and from early childhood to adolescence [16] but to our knowledge none have assessed the wide age-range studied here. Adult MRI brain atlases perform sub-optimally when applied to infant brain images due largely to the global difference in size between adult and infant brains. In addition, the relative size of brain regions also changes during development due to non-uniform brain growth [38]. This means that the regional boundaries on an adult brain atlas may not accurately correspond to the same regions on a young brain, leading to inaccurate labelling and segmentation of infant brain regions. These issues are especially problematic for longitudinal studies, where the degree of distortion will change as the transformations needed decrease with increasing age and brain size. Our developmental atlas minimises these issues by providing registration templates at different stages of development, so that global and regional size difference between atlas and subject images are reduced. We envisage future studies into neonatal white matter development registering their images to the mean FA template that most closely matches their sample age.

## Conclusion

In summary, we describe reproducible protocols for tractography in extremely premature neonates and infants, and report developmental trends for diffusion metrics throughout this developmental period. We have shown tract-specific white matter development from 25 weeks PMA to 2 years of age, and show that white matter tracts mature at different rates in early life, corroborating previous histological findings *in vivo*. We have also produced the first registration template that covers the period from 25 weeks PMA to 2 years of age. The atlas is freely available at www.brain-development.org.
